# Supplementary material for: The structure of iPLA2β reveals dimeric active sites and suggests mechanisms of regulation and localization
Source: Nat Commun. 2018 Feb 22;9:765. doi: 10.1038/s41467-018-03193-0 (PMC5823874; doi:10.1038/s41467-018-03193-0)
Supplement: Supplementary file 1 — Supplementary Information [file 41467_2018_3193_MOESM1_ESM.pdf]

The structure of iPLA$_2$β reveals dimeric active sites and suggests mechanisms of regulation and localization

Malley et al.

Supplementary Information

Supplementary Table 1. Data collection and Refinement statistics

Crystal	Native	SeMet	SeATP
Wavelength (Å)	0.98	0.9794	0.9793
Resolution range	39 - 3.95	50-4.08	30-4.6
Space group	P 6_2	P 6_2	P 6_2
Unit cell (Å)	266.1 266.1 79.4	270.3 270.3 79.7	270.6 270.6 79.3
Angles (°)	90 90 120	90 90 120	90 90 120
Total reflections	177963	123675	122724
Unique reflections[1,2]	28492 (1414)	23935(785)	17975 (789)
Multiplicity	6.2	5.2	6.8
Completeness (%)[2]	100 (100)	89.3 (58.5)	97.1 (88.2)
Mean I/sigma(I) [2]	17 (1.6)	14.6 (1.2)	12.75 (0.8)
Wilson B-factor	39.6		
R-merge[2]	0.101 (1.08)	0.084(0.73)	0.118(1.19)
R-pim[2]	0.044 (0.46)	0.038(0.37)	0.044(0.58)
CC1/2[3]	0.70	0.64	0.48
Xtriage reflections[4]	24660	20087	14873
Xtriage completeness (%)[4]	86(75)	75(54)	76(15)
Xtriage anomalous completeness(%)[4]		65	72
Xtriage effective resolution: overall d_min and d_min along a*, b* and c* (Å) [4]	3.95; 4.43; 4.43; 3.97	4.08; 4.68; 4.68; 4.19	4.62; 5.45; 5.45; 4.66
Reflections used in refinement[2]	24658 (906)		
Reflections used for R-free[2]	2461 (82)		
R-work[2]	0.26 (0.25)		
R-free[2]	0.31 (0.28)		
Number of TLS regions	6		
Number of non-	9396		

hydrogen atoms	
Protein residues	1236
RMS(bonds)	0.005
RMS(angles)	1.07
Ramachandran favored (%)	83.5
Ramachandran allowed (%)	16.1
Ramachandran outliers (%)	0.4
Rotamer outliers (%)	0.00
Clash score	20.6
Average B-factor	67.57

[1]HKL2000 scaling statistics reported for all processed reflections before rejection with "autocorrection" function (see below footnote 4).

[2]Statistics for the highest-resolution shell are shown in parentheses: (4.0-3.95 Å), (4.15-4.08), (4.68-4.60) for Native, SeMet and SeATP data, correspondingly and (4.09 – 3.95) for refinement.

[3]CC1/2 is shown only for the highest-resolution shells defined in footnote 2.

[4] Statistics reported by Xtriage program reflecting strong anisotropy and rejection of weak reflections after scaling with "autocorrection" option in HKL2000. High resolution shells reported in Xtriage output are (5.16-3.95 Å) for native data, (5.21-4.08 Å) for SeMet data, (5.08-4.61 Å) for SeATP data.

Supplementary Figure 1.

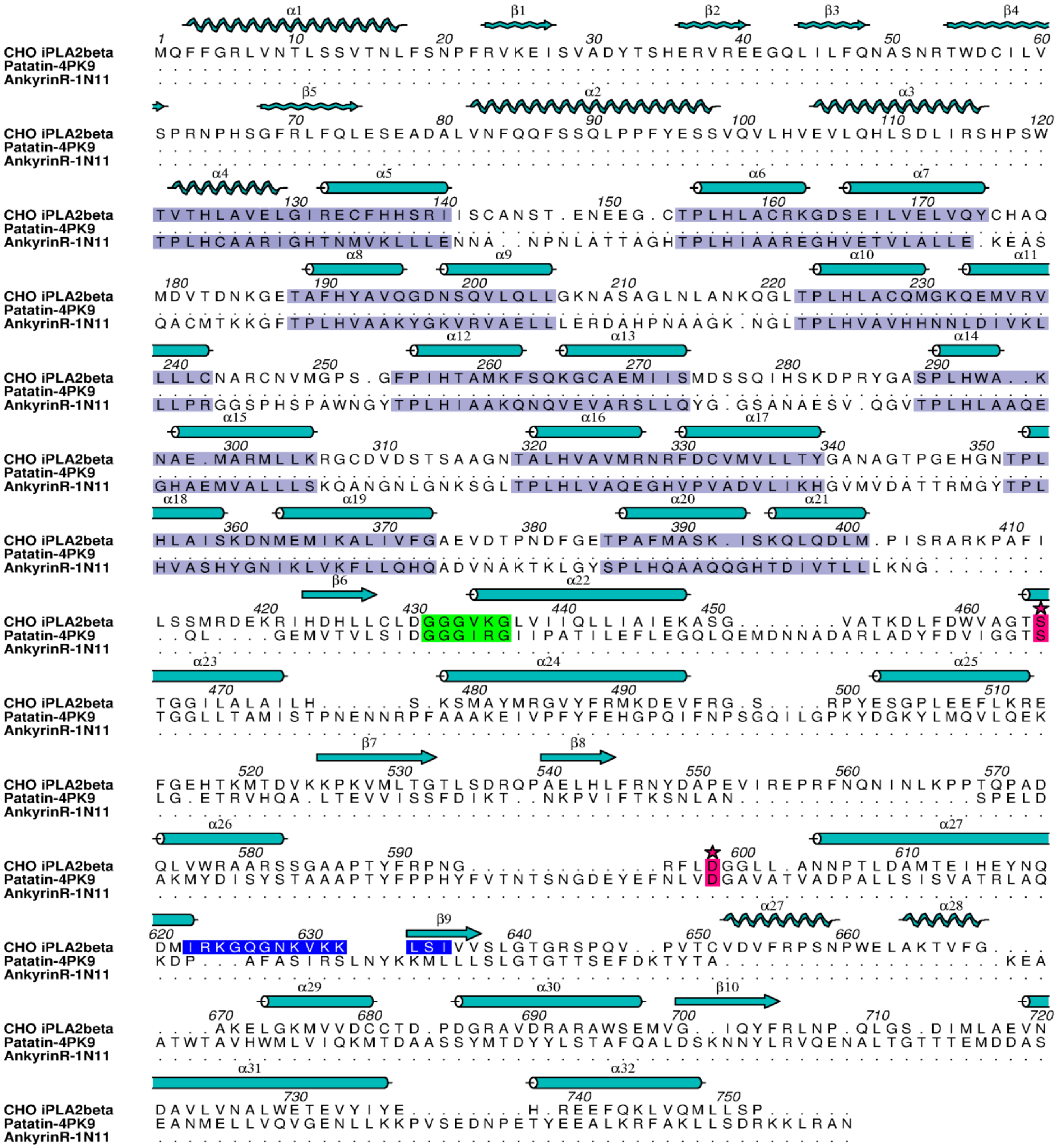

Structure-based alignment of the iPLA$_2$β amino acid sequence with patatin (PDB: 4PK9) and the eight ankyrin repeats of ankyrin-R (PDB: 1N11) using TM-align (https://zhanglab.ccmb.med.umich.edu/TM-align/). The ARs are highlighted in light purple, the active site residues starred in magenta, the oxyanion hole in green, and the 1-9-14 CaM-binding motif in blue. Helices and sheets from the structure are depicted with cylinders and straight arrows, respectively, and predicted secondary structure elements in the N-terminus and membrane interacting regions are shown with wavy lines and arrows.

Supplementary Figure 2. **Representative electron density maps**.

a)

b)

c)

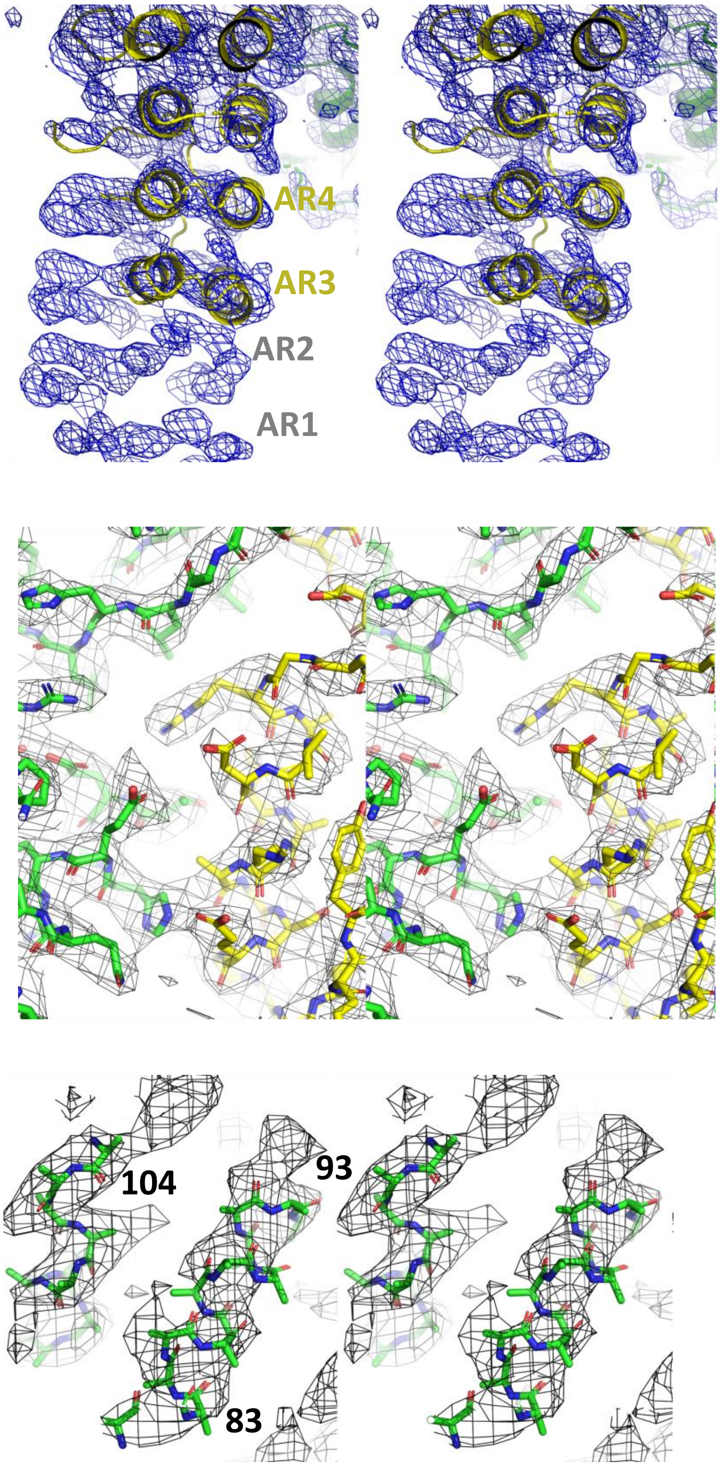

a) Stereo view of the electron density map calculated with MR/SAD solution using coefficients from *overall_best_denmod_map_coeff.mtz* at 1.5 σ contour level around ANK domain. MR model used in calculation is shown by yellow cartoon representation. AR1 and -2 and loops of AR3, and -4 are not modeled. **b)** Electron density map calculated with 2Fo-Fc coefficients and contoured at 2 σ level for the final refined model representing part of the dimerization interface. **c)** Same electron density map around N-terminal helixes contoured at 1 σ level.

Supplementary Figure 3. Comparison of patatin and iPLA$_2$β CAT domains.

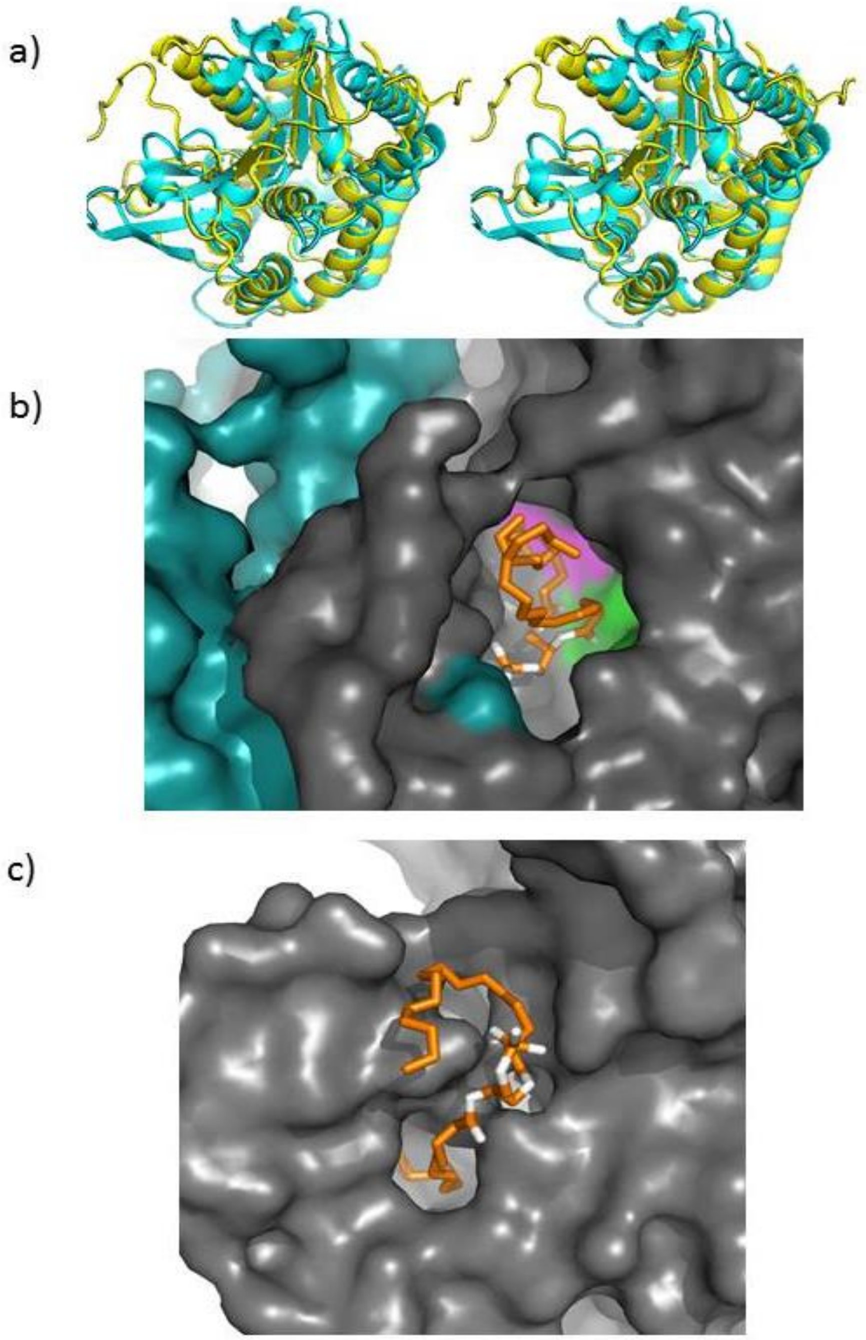

a) Stereo view of the superposition of the iPLA$_2$β catalytic domain shown in yellow with patatin (PDB ID:4PK9), shown in cyan; **b)** An open conformation of the active site of iPLA$_2$β. The surface representation of the iPLA$_2$β active site region (the view from the membrane) is shown in grey for monomer A and in cyan for monomer B. Active site residues are highlighted in green and magenta. To illustrate the open conformation and accessibility of the active site, the 1,2-dipalmitoyl-3-sn-phosphatidylethanolamine molecule shown with stick representation in orange and white was inserted using the Dock program. **c)** Surface representation of the membrane-interacting region of patatin in similar orientation. Catalytic residues are completely inaccessible and only one fatty acid chain can be fitted into narrow cavity leading to the active site.

Supplementary Figure 4.

a)

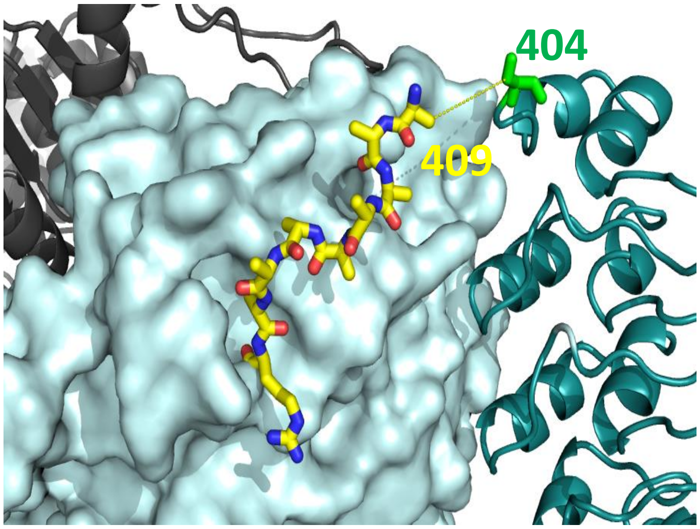

b)

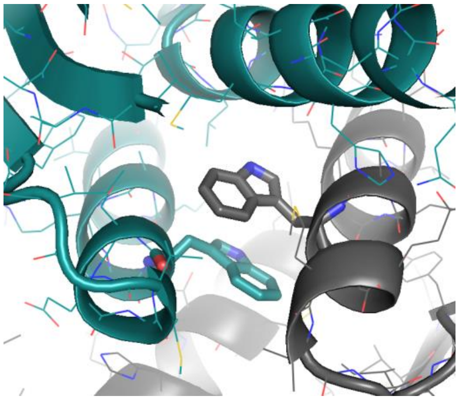

c)

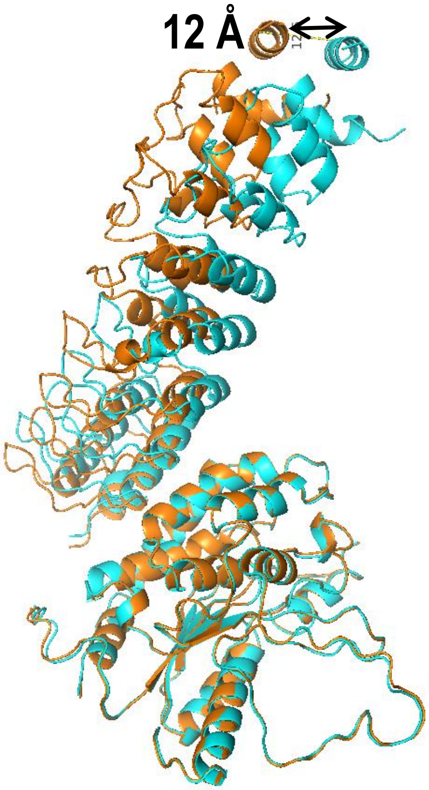

a) Conformation of partially resolved loop, shown in yellow, red and blue stick representation, connecting CAT and ANK domain. CAT domain is shown in surface representation and ANK in cartoon representation. **b)** Interactions of Trp695 shown in stick representation in a dimerization interface. One molecule is shown in grey and the other in dark cyan. **c**) Movement of ANK upon superimposition of CAT domains. One molecule is colored in orange and the other in cyan.

Supplementary Figure 5. Detection of the ATP-binding site.

a)

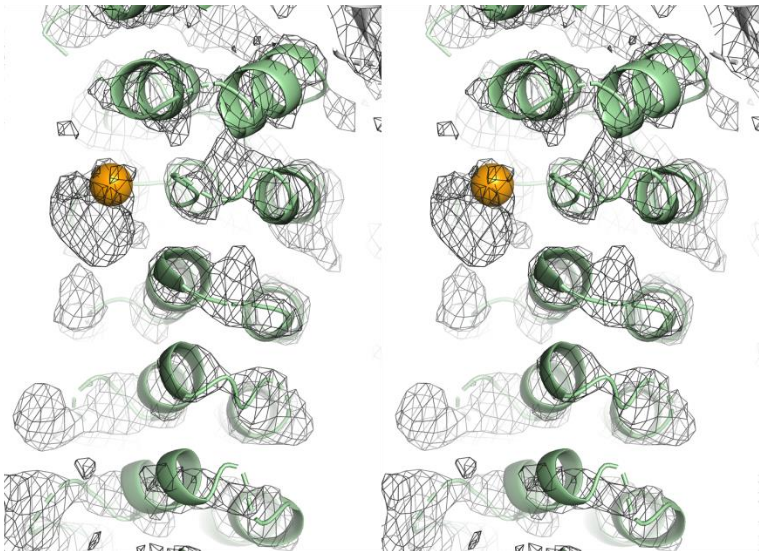

b)

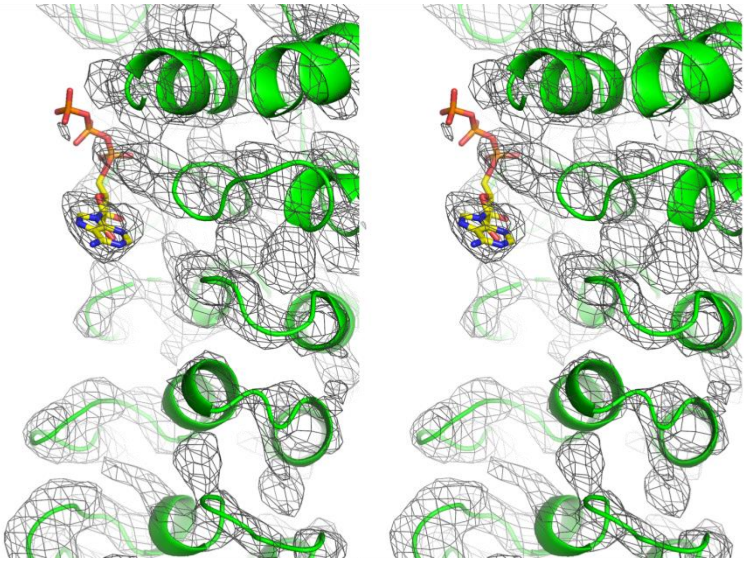

a) Stereo view of anomalous peak (orange sphere) found in MR/SAD solution with data collected from Se-ATP soaked crystal and of the experimental electron density map calculated using coefficient from *overall_best_denmod_map_coeff.mtz* file and contoured at 1.5 s level. ANK domain is shown in slate cartoon representation. **b**) Fitting of ATP shown in stick representation into MR/SAD electron density map of the SeMet data. ANK domain is shown in green cartoon representation.

Supplementary Figure 6. iPLA$_2$β dimerization in solution.

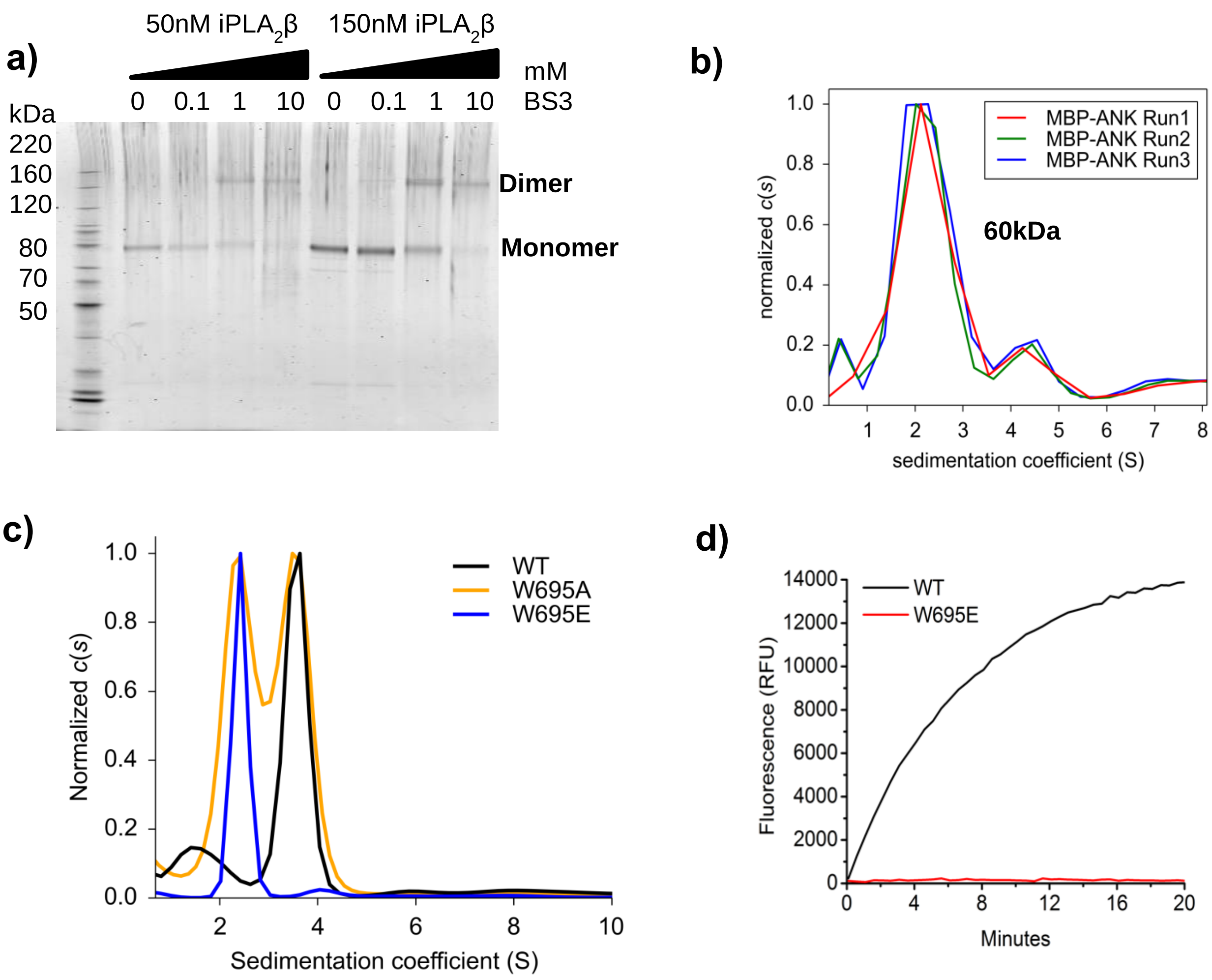

a) Dimerization of iPLA$_2$β revealed by crosslinking experiments. The protein was treated with increasing concentrations of BS3 amine-reactive crosslinking agent (concentration in mM is shown above the gel) for 1 hour. The reaction was quenched by Tris-glycine loading buffer, proteins were separated by SDS PAGE with Invitrogen Benchmark protein ladder, stained with Krypton fluorescent protein stain, and visualized at 523 nm excitation and 580 nm emission on a Typhoon imager. **b)** C(s) distribution of purified MBP-tagged ANK domain (177-389) from three independent sedimentation velocity experiments. The estimated molecular weight corresponds to a monomer of the MBP-ANK (average MW of peaks is 60kDa versus theoretical 63 kDa). **c)** C(s) distribution of wild type (black), W695E (blue) and W695A (orange) mutants from sedimentation velocity experiment performed as described for Fig. 4a. **d)** Phospholipase activity (see below Supplementary Figure 7 a-e) of WT and W695E mutants. Results are representative of three independent experiments.

Supplementary Figure 7. Enzymatic activity and inhibition measured with pyrene-PC substrate.

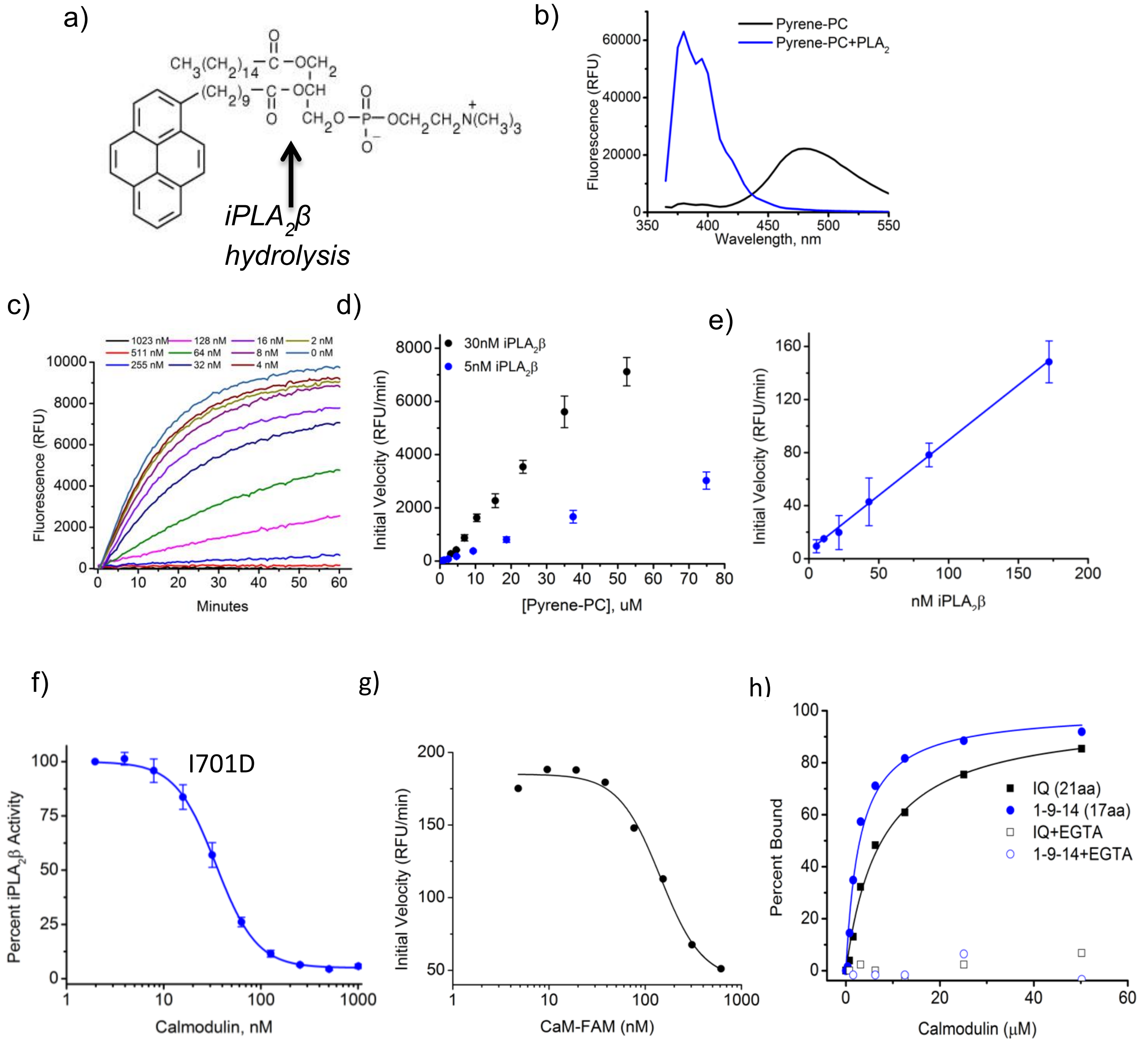

a) Structure of pyrene-PC. **b)** Fluorescence spectra at 342 nm excitation of the substrate before and after cleavage. **c)** Real time measurements of pyrene-PC cleavage by $iPLA_2\beta$ in the presence of different concentrations of CaM. RFU – relative fluorescence unit. The initial slope of these data was used in preparing Fig 4c. **d)** Plots of initial velocity vs. substrate concentration at two different concentrations of enzyme. **e)** Validation of the assay demonstrating the linear relationship between initial velocity and the enzyme concentration. Error bars represent average ± s.e.m of assays performed in triplicate. **f)** Calmodulin inhibition of the I701D mutant. **g)** Inhibition of $iPLA_2\beta$ by FAM-CaM. **h**) Binding of IQ and 1-9-14 FAM-labeled synthetic peptides to CaM measured by fluorescence anisotropy. Data is representative of three independent experiments.
